# Influence of ball milling parameters on the mechano-chemical conversion of polyolefins†

**DOI:** 10.1039/d4mr00098f

**Published:** 2024-12-18

**Authors:** Adrian H. Hergesell, Claire L. Seitzinger, Justin Burg, Renate J. Baarslag, Ina Vollmer

**Affiliations:** a Inorganic Chemistry and Catalysis, Institute for Sustainable and Circular Chemistry, Utrecht University Universiteitsweg 99 3584 CG Utrecht The Netherlands i.vollmer@uu.nl

## Abstract

Ball-milling of addition polymers such as polyolefins, polystyrene and polyacrylates can be used for depolymerization to obtain the respective monomers. However, absolute yields are typically low, especially from polyolefins which are notoriously difficult to depolymerize. To increase the viability of ball milling as a recycling technique, the effect of milling parameters on small hydrocarbon and monomer yields has to be understood. Herein, we systematically investigate the influence of sphere material, milling frequency, plastic filling degree, and milling temperature. Heavy spheres and high milling frequencies boost hydrocarbon yields by maximizing mechanical forces and frequency of collisions. While the dose of kinetic energy is commonly used to describe mechano-chemical processes, we found that it does not capture the mechano-chemical depolymerization of polyolefins. Instead, we rationalized the results based on the Zhurkov equation, a model developed for the thermo-mechanical scission of polymers under stress. In addition, low plastic filling degrees allow for high percentage yields, but cause significant wear on the grinding tools, prohibiting sustained milling. Milling below 40 °C is beneficial for brittle chain cleavage and depolymerization. This study provides a new approach to rationalize the influence of individual milling parameters and their interplay and serves as a starting point to derive design principles for larger-scale mechano-chemical depolymerization processes.

## Abstract 2

Mechano-chemical plastic recycling suffers from low overall yields. We identified the role of key parameters to maximize mechanical impact to increase the productivity of small hydrocarbons, such as propene, from polypropylene.

## Graphical abstract

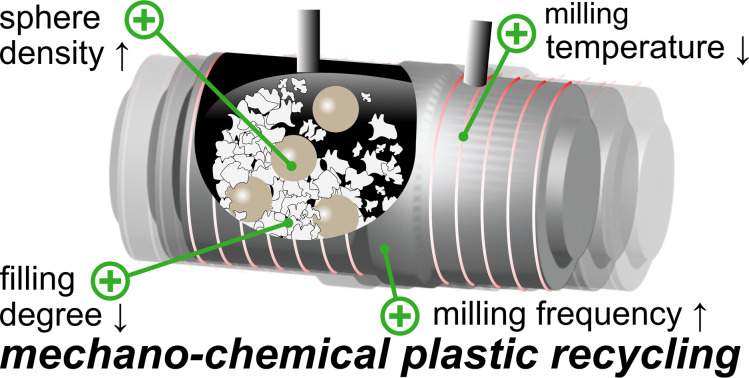

## Introduction

Most plastic waste is not managed sustainably. Incineration leads to the emission of dangerous gases and CO_2_, while landfilling causes leakage of harmful chemicals and micro- and nanoplastics.^1,2^ While lower plastic production rates must relieve the pressure on waste management systems and the environment in the future, recycling can enable a more sustainable end of life for remaining plastic production.^3^ Besides legislative efforts, suitable robust technologies can help create economic incentives to increase the recycling rate, which is only 14% by weight currently.^4^ However, common mechanical recycling, *i.e.*, melting and reshaping of plastic items, deteriorates material properties.^5,6^ Recent developments have focused on chemical recycling to convert plastic waste to chemicals, such as monomers for the production of pristine plastics, that can replace fossil raw materials in the chemical industry.^7–12^ Current chemical recycling efforts mostly rely on pyrolysis, heating the plastic in an inert atmosphere to induce cracking.^7^ However, this high-temperature process is energy-intensive and yields product mixtures with a low value. Especially for polyethylene (PE) and polypropylene (PP), the most produced plastics globally, hundreds of different hydrocarbons are obtained.^13^ The high temperatures required to break stable C–C bonds in the backbone of PE and PP also promote less selective follow-up reactions of highly reactive intermediates, thus prohibiting precise control over the product distribution. In contrast, low-temperature approaches promise higher selectivity and energy efficiency.^14^

Exposing PP to mechanical forces can lead to backbone cleavage.^15^ In this mechano-chemical pathway, mechanical strain lowers the vibrational energy needed for covalent bond scission, and the bond is not broken by thermal energy alone.^16^ Chain cleavage processes *via* thermo-mechanical activation can be described using the Zhurkov equation (eqn (1)). It is based on the Arrhenius equation and describes a rate constant for chain cleavage *k* in dependence of a stress *σ*, where *k*_0_ is the preexponential factor, *E*_A_ is the activation energy barrier, and *α* is the activation volume, a constant translating macroscopically applied stress to the microscopic force to which the bond is exposed.^14,17^1
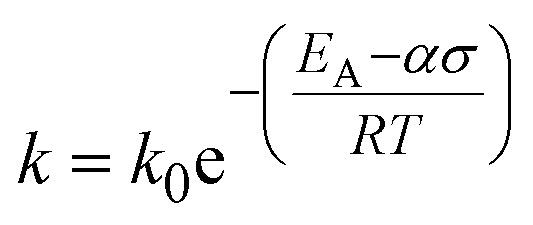


While mechano-chemical chain scission is unwanted in polymer production, it has recently been exploited for purposeful chain cleavage and the recycling of plastic materials.^14^ Ball milling has been reported to enable the conversion of polystyrene,^18,19^ poly(α-methyl styrene),^20^ and polymethacrylates^21^ to their monomers. The reported mechano-chemical depolymerization of such polymers proceeds *via* radical intermediates.^14,18–20^ The initiation step is the backbone cleavage of polymer chains and the generation of a pair of mechano-radicals. These can then undergo direct depolymerization to the monomer *via* β-scission, or participate in other follow-up reactions, including H-abstraction, disproportionation and combination, giving rise to other products besides monomers.^14^ The reported polymers undergo facile depolymerization for two reasons: (i) they have high glass transition temperatures *T*_g_, enabling brittleness and facile chain cleavage at ambient conditions which generates radical intermediates.^22^ In addition they (ii) have low ceiling temperatures *T*_c_, providing good thermodynamic driving forces for depolymerization of these radical intermediates. Therefore, both *T*_g_ and *T*_c_ have to be sufficiently high and low, respectively, in order to enable efficient chain cleavage and depolymerization. In contrast, PE and PP with a low *T*_g_ and a high *T*_c_ are very difficult to convert,^14,23^ and their additive-free non-catalytic depolymerization to monomers *via* mechano-chemistry has not yet been studied systematically.

While the applicability of mechano-chemical recycling has been showcased for a variety of polymers with a C–C backbone, efforts to scale up this approach are dependent on achieving high yields. In contrast, reported absolute yields are notoriously low and in the range of milligrams. High relative percentage yields based on the amount of starting material are often reached only in specific combinations of high milling frequencies and low polymer loadings, *i.e.*, plastic filling degrees, in the ball milling vessel.^20,21^ While these experiments strikingly showcase the technology's potential, the suitability of the parameters studied is questionable in an industrial setting. A deeper understanding of the influence of milling parameters and their interplay with plastic material characteristics is necessary to optimize the process and achieve high absolute yields and good selectivities, especially for the most abundant and difficult-to-depolymerize PE and PP. To this end, we herein systematically investigate the influence of the milling parameters sphere material and number, milling frequency, plastic filling degree, and milling temperature on the production of small hydrocarbons during mechano-chemical recycling of polyolefins and develop a kinetic model based on the Zhurkov equation.

## Results and discussion

**Scheme 1:**
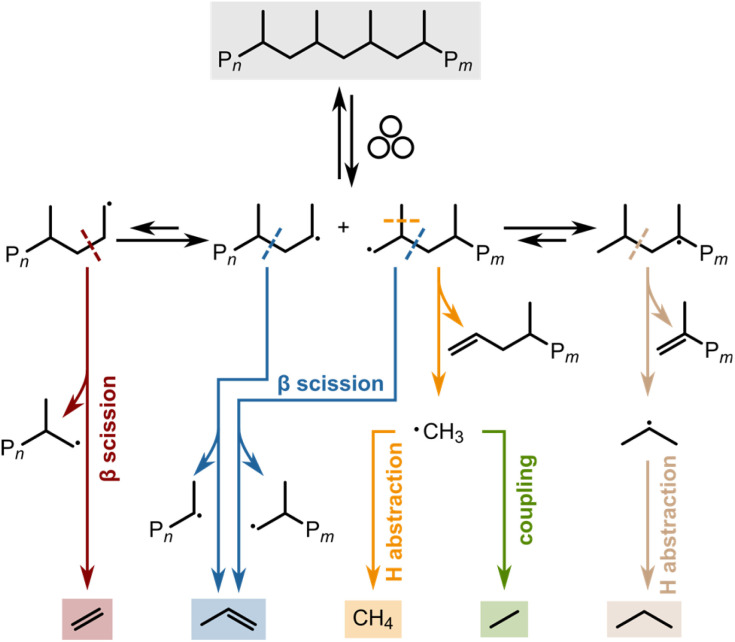
Proposed examples of mechanistic steps *via* which C_1–3_ hydrocarbons are generated during ball milling of PP. Mechano-chemical backbone scission forms a primary and a secondary radical which can undergo subsequent radical transfer, hydrogen abstraction, scission, and coupling reactions to form methane, ethane, ethene, propane, and propene.

We used a Retsch shaker mill to mechano-chemically depolymerize PP and PE (see Section S1† for experimental details and Table S1† for plastic characteristics). We have recently reported on the depolymerization of PP *via* ball milling.^24^ Mechanistically, backbone cleavage leads to the formation of mechano-radicals which can undergo a variety of hydrogen transfer, scission, and coupling reactions leading to C_1–3_ hydrocarbons (Scheme 1). To allow for the online analysis of gaseous hydrocarbon products, we equipped a commercial container with gas connections. Products were eluted using a steady stream of nitrogen and analyzed using an online gas chromatograph. The continuous removal of products drives the depolymerization equilibrium according to Le Chatelier's principle, countering the adverse thermodynamics of low-temperature depolymerization.^25^ We focus our analysis on gaseous hydrocarbons (C_1–3_), especially propene, the monomer of PP. Small hydrocarbons are ideal for time-resolved analysis due to their volatility and short residence time in the reactor setup. Furthermore, PP materials often contain heavier additives, such as stabilizers or residual heavier hydrocarbons, which could interfere with the analysis of hydrocarbons generated *via* mechano-chemical degradation.^26^

### Role of sphere density

**Fig. 1:**
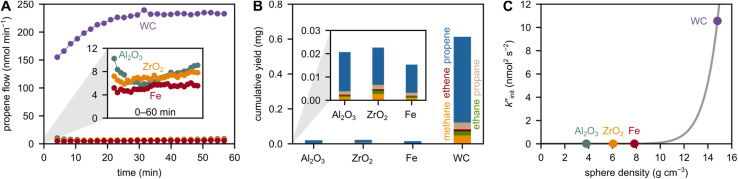
Influence of grinding sphere density on small hydrocarbon production. (A) Propene flow during milling of 2 g model PP at 30 Hz with 5 Al_2_O_3_, ZrO_2_, Fe, and WC grinding spheres (10 mm diameter). The inset shows a magnification of the propene flow. (B) Cumulative C_1–3_ hydrocarbon yields obtained after 1 h of milling 2 g model PP at 30 Hz with 5 Al_2_O_3_, ZrO_2_, Fe, and WC grinding spheres (10 mm diameter). The inset shows a magnification of the cumulative yield. (C) 
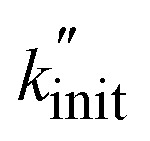
as a measure for chain cleavage calculated from experimental data *via*eqn (15) plotted against grinding sphere density *ρ* and fit (gray line) using the modified Zhurkov equation (eqn (12)).

To investigate the role of the grinding sphere material, we used 5 grinding spheres with a diameter of 10 mm at 30 Hz. We tested grinding spheres made from alumina (Al_2_O_3_), zirconia (ZrO_2_), and tungsten carbide (WC), besides metallic grinding spheres made from stainless steel (Fe). The densities of these spheres are considerably different, influencing the force exerted on the plastic material (Table S2,†Fig. 1A). The main product for all spheres is propene, besides methane, ethane, ethene and propane (Fig. 1B, see Fig. S1† for milling of PE). WC with the highest density produces the highest amount of propene. The stark differences in small hydrocarbon productivity can be rationalized in terms of different rates of backbone cleavage, which is the initiation step in mechano-chemical depolymerization of polyolefins.

In the following, we develop a simplified model to understand the observed exponential increase in gas phase product formation with sphere density. The impact kinetic energy *E*_kin_ of a grinding sphere is described by eqn (2), where *m* is the mass, and *ν* the impact velocity of a grinding sphere. The mass of a grinding sphere is expressed as a function of its radius *r* and material density *ρ*.2
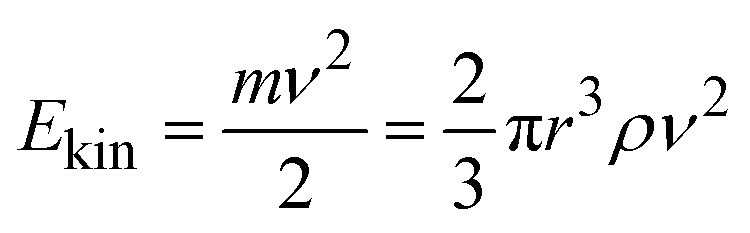


The dose of kinetic energy (DKE) supplied to the system in a given time *t* is given in eqn (3) where *E*_kin_ is the kinetic energy, and *ṅ*_coll_ the collision rate. The prerequisite for the applicability of this equation is that *E*_kin_ and *ṅ*_coll_ are well-defined values and constant over time.

3 DKE = *E*_kin_*ṅ*_coll_*t*

To understand the dependency of DKE on the grinding sphere density *ρ*, we have to understand the dependencies of *E*_kin_ and *ṅ*_coll_ on *ρ*. To understand the dependency of *E*_kin_ on *ρ*, we assume that the grinding sphere velocity *ν* is independent of the sphere material. This is based on the following argument: when the milling is started, the spheres are at rest and then are accelerated by the container. The magnitude of the acceleration is independent of the sphere mass/density, but only depends on the set shaking frequency to be reached by the container. As the spheres travel the same distance, the length of the container, their final velocity will be the same for spheres of different densities. Upon impact, two limiting cases can be considered, which lead to the same result. Assuming (i) elastic collisions, the sphere's momentum and velocity are either conserved or modified in the same manner, regardless of the material density. For example, the magnitude of the rebound velocity vector of a grinding sphere after elastic contact with an infinitely heavy wall is the same as its incoming velocity due to conservation of momentum, regardless of its material density. Assuming (ii) inelastic collisions, the grinding spheres are stopped at the container wall and subsequently accelerated to maximum container velocity again by the moving container. Hence, due to limiting cases (i) and (ii), we assume that the trajectories of grinding spheres in the container are independent of the material. Therefore, because *ν* is independent of the sphere material and *m* is proportional to *ρ*, the kinetic energy of a sphere with a certain size scales linearly with its density (eqn (2)). In reality, deviations from this idealized case are expected since the nature of collisions might not be sufficiently captured by the elastic and inelastic boundary cases, which might add complexity to the acceleration/deceleration behavior of grinding spheres. In addition, the acceleration/deceleration behavior might be dependent on material density. However, for the sake of simplicity, we for now assume the validity of our assumptions.

To understand the dependency of collision frequency *ṅ*_coll_ on *ρ*, we assume again that the trajectories of grinding spheres and therefore their collision rates are independent of the material. Therefore, *ṅ*_coll_ should also be independent of the material and its density *ρ*.

With *E*_kin_ ∝ *ρ* and *ṅ*_coll_ independent of *ρ* we derive that the DKE should scale linearly with *ρ*. This is in line with values calculated according to Jafter *et al.*,^27^ where milling with WC *vs.* Al_2_O_3_ supplies a total energy of 165.6 *vs.* 42.5 kJ to the system within one hour (Table S3†).

Yields in mechano-chemical milling have been connected to the DKE in the past, and linear relationships have been observed, especially in well-mixed powder systems.^28,29^ However, the hydrocarbon yields observed by us clearly do not show this relationship, having an exponential rather than linear dependency on the grinding sphere density (Fig. S2†). Jafter *et al.* likewise acknowledged that the total energy approach is not suitable to predict yields during milling of polymers.^27^

The kinetic energy that is supplied to the system is partly dissipated as heat, and the formation of small and short-lived energy-rich environments which are generated upon impact have been discussed in the mechano-chemical literature as “hot spots”.^30,31^ While these entities could in principle accelerate reactions thermally, we believe that this phenomenon does not fully capture the reactivity of polymers under mechanical impact.^32^ Xie *et al.*^33^ have performed molecular dynamics simulations on polyethylene, and found that shock loads of >1000 m s^−1^ are necessary to reach cracking temperatures, while grinding sphere velocities in standard mixer mills are commonly <10 m s^−1^.^27,28,30^

Instead of relying on “hot spots”, we adapted the Zhurkov model (eqn (1)), developed for tensile test experiments under stress, to ball milling. Increasing the density of the grinding spheres mainly changes the stress which the material is exposed to. We derive the stress *σ* needed for the Zhurkov equation using the Hertz contact theory^34,35^ according to eqn (4), where *F*_impact_ describes the normal force exerted by the milling sphere on the polymer agglomerate and is defined according to eqn (5). Here, *m* and *r* are the sphere mass and radius as used in eqn (2), *a*_max_ is the maximum acceleration as derived in Section S2,† and *A*_impact_ is the radius of impact defined according to eqn (6).^36^4
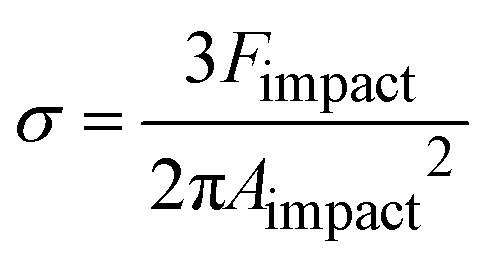

5 *F*_impact_ = *ma*_max_

6
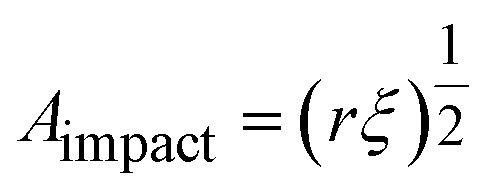


We derive *ξ*, the depth of indentation, using the definition of the normal force according to Hertz theory. The normal force during indentation of a sphere on an elastic surface is given by eqn (7), where E* is the reduced Young's modulus (see below), and by rearranging, we obtain eqn (8).^34^7
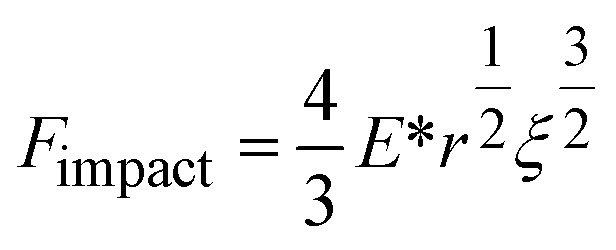
8
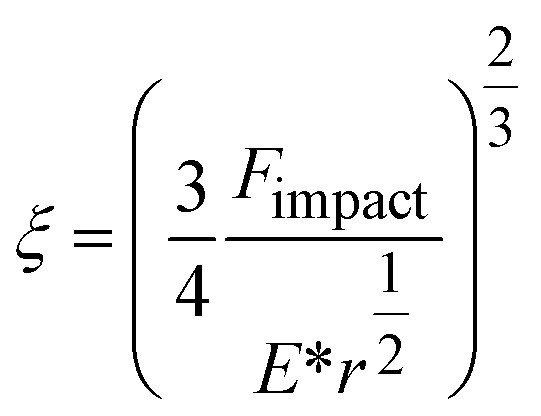
In eqn (4) we substitute *A*_impact_ using eqn (6). In addition, we substitute *F*_impact_ using eqn (7), where we use eqn (8) as an expression for *ξ*. This yields eqn (9). Now, using eqn (5) for *F*_impact_ and the derivation of *a*_max_ according to Section S2,† together with an expression for the grinding sphere mass in terms of its radius *r* and its density *ρ*, we obtain eqn (10).9
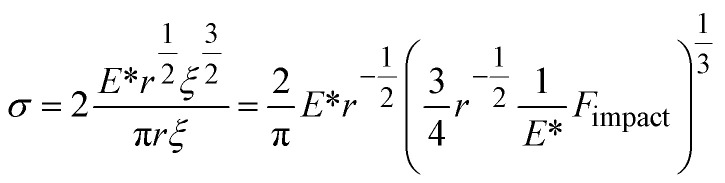
10
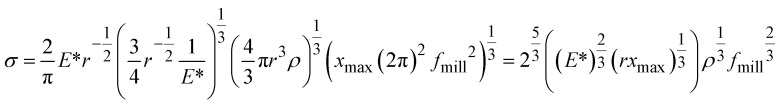


To describe the interaction of different materials during contact, the reduced Young's modulus E* can be used according to eqn (11), where *E*_1,2_ and *μ*_1,2_ are the Young's moduli and Poisson's ratios of materials 1 (spheres) and 2 (PP), respectively (Table S2†).^34^E* is dictated by the very low Young's modulus of PP, and therefore largely independent of the material characteristics of the grinding spheres.11
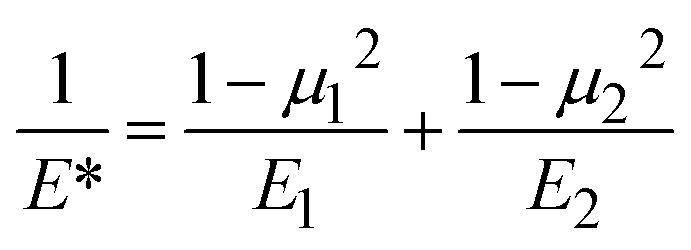


To understand the mechano-chemical activation of PP on a molecular level, we express the stress as 
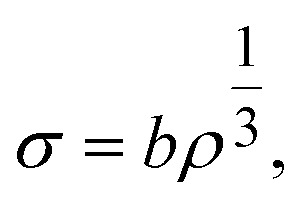
 with *b* being the proportionality constant, according to eqn (10). The Zhurkov equation (eqn (1)) can then be rewritten, assuming constant temperature (eqn (14)), with the definitions in eqn (12) and (13). This equation shows the heavy dependency of chain cleavage rate *k* on sphere density *ρ*.12
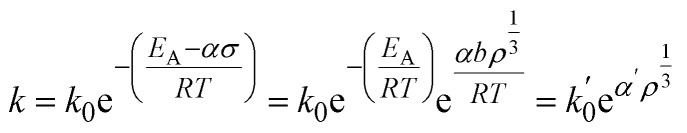
13
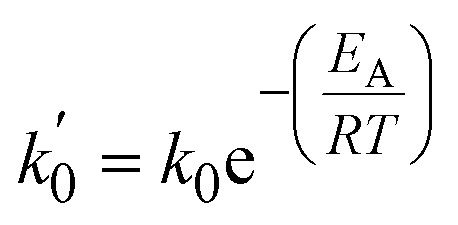
14
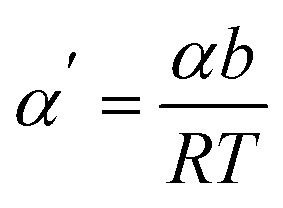


To derive a measure for *k* from experimental product distributions, we use a steady state approximation, and a simplified reaction system consisting of initiation (homolytic chain cleavage), β-scission to produce propene (depolymerization step), and termination *via* combination or disproportionation. We derive 
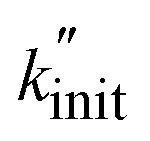
 (eqn (15), see Section S3† for derivation) as a metric for chain cleavage events, where *n*(*M*) is the number of monomer molecules generated during the milling time *t*.15
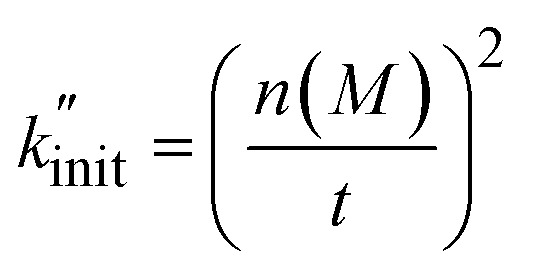


This 
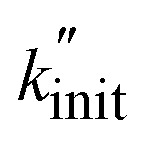
 can thus be used as a suitable metric to assess the influence of higher sphere densities, when plotting 
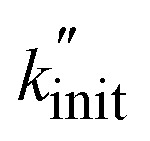
*vs. ρ*. The observed trends in hydrocarbon productivity are captured by the exponential relationship (eqn (12)), which predicts much higher chain cleavage rates and hydrocarbon yields when milling with heavy WC compared to lighter Fe, ZrO_2_, or Al_2_O_3_ spheres (Fig. 1C). According to the modified Zhurkov equation, milling with heavier grinding spheres increases the macroscopic stress and thereby aids backbone cleavage and subsequent depolymerization.

However, the density of grinding spheres is not the only descriptor for small hydrocarbon productivity. This is especially evident when comparing the lighter grinding spheres made from ZrO_2_, Al_2_O_3_ and Fe. The densities in this group are *ρ* (Al_2_O_3_) < *ρ* (ZrO_2_) < *ρ* (Fe), and a similar trend in small hydrocarbon productivity could be expected. However, this trend is not observed. Milling with Fe produces less hydrocarbons than milling with Al_2_O_3_ or ZrO_2_ (see insets in Fig. 1). Despite the density, other mechanical properties such as Young's modulus (*E*) and Poisson's ratio (*μ*) could play some role for local forces and pressures in contact mechanics, according to Hertz theory.^37,38^ However, ZrO_2_ and Fe have very similar *E* and *μ* values, and classical contact mechanics do not seem to capture the difference in their hydrocarbon productivities. We preliminarily ascribe the gap in mechano-chemical activities to differences in surface chemistry, rather than purely contact mechanical reasons. For instance, metal oxides are known to possess oxygen vacancies and can contain partially reduced sites. These defects could interact with and stabilize radical intermediates during milling better than Fe could, thereby driving mechano-chemical depolymerization beyond pure force maximization.

### Role of milling frequency

**Fig. 2:**
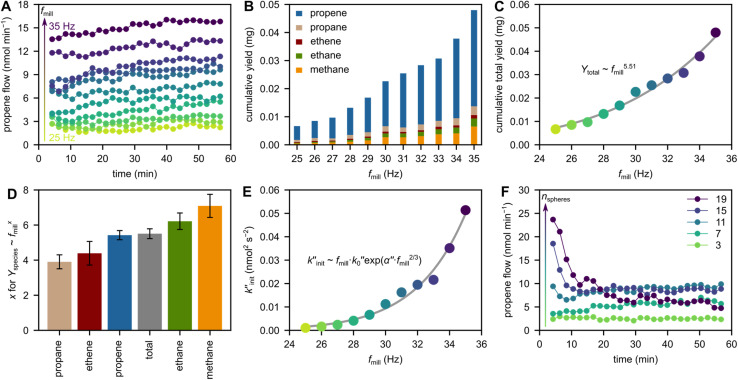
Variation of milling frequency and sphere number. (A) Propene flow during milling of 2 g model PP at 25–35 Hz with 5 ZrO_2_ grinding spheres (10 mm diameter). (B) Cumulative C_1–3_ hydrocarbon yields obtained after 1 h of milling 2 g model PP at 25–35 Hz with 5 ZrO_2_ grinding spheres (10 mm diameter). (C) Cumulative total C_1–3_ hydrocarbon yields (*Y*_total_) obtained after 1 h of milling 2 g model PP at 25–35 Hz with 5 ZrO_2_ grinding spheres (10 mm diameter), and fit (*Y*_total_ ∼ *f*_mill_^5.51^). (D) Dependency of hydrocarbon formation on *f*_mill_: exponent *x*_*i*_ obtained by fitting *Y*_*i*_ per mg ∝ (*f*_mill_ per Hz)^*x*_i_^ for each product *i*. *Y*_*i*_ denotes the cumulative yield of *i* after 1 h with 5 ZrO_2_ grinding spheres (10 mm diameter) at *f*_mill_ = 25–35 Hz. (E) 
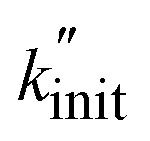
 as a measure for chain cleavage calculated from experimental data *via*eqn (15) plotted against milling frequency *f*_mill_ together with fit (gray line) using the modified Zhurkov equation (eqn (16)). (F) Propene flow during milling of 2 g model PP at 30 Hz with 3–19 ZrO_2_ grinding spheres (10 mm diameter).

To investigate the role of shaking frequency *f*_mill_, we milled 2 g model PP with 5 ZrO_2_ grinding spheres at frequencies between 25 and 35 Hz. Milling at higher frequency increases the observed propene flows (Fig. 2A) and cumulative C_1–3_ hydrocarbon yields (Fig. 2B). This general trend is rationalized by the higher velocity and kinetic energy of grinding spheres when milling at higher frequency, which leads to more backbone cleavage, and is thus reflected in the production of small hydrocarbons.

The kinetic energy of a grinding sphere *E*_kin_ is given by eqn (2), and the impact velocity *ν* of grinding spheres is dependent on *f*_mill_. During shaking, the grinding spheres are accelerated due to contact with the milling container, and we assume that *ν* is equal to the maximum velocity of the milling vessel and therefore proportional to *f*_mill_ (see Section S2† for derivation). Shaking with a single grinding sphere has been analyzed *via* high-speed videos and discrete element models and also suggests a linear dependency of *ν* on *f*_mill_.^28,30^ The same holds for the collision frequency *ṅ*_coll_ of a single grinding sphere with the reactor wall, which is likewise linearly dependent on *f*_mill_.^28,30^ Assuming that both *ν* and *ṅ*_coll_ are proportional to *f*_mill_, the DKE can be estimated according to eqn (3). Since *E*_kin_ and *ṅ*_coll_ are then expected to scale with *f*_mill_^2^ and *f*_mill_, respectively, the proportionality DKE ∝ *f*_mill_^3^ is expected.

A linear dependency of the depolymerization yield *Y* on DKE would dictate *Y* ∝ *f*_mill_^3^. In our case, however, the increase of product formation with milling frequency is much more pronounced. Instead, overall hydrocarbon yield from PP scales with *f*_mill_^5.51^ (Fig. 2C). Propene formation scales with *f*_mill_^5.42^, which is close to the overall yields due to propene being the main product. Interestingly, different products show different sensitivities towards a variation in frequency (Fig. 2D, see Fig. S3† for individual fits). Propane yields, for instance, scale with *f*_mill_^3.91^ while methane productivity scales with *f*_mill_^7.09^. We believe that these differences are due to the mechanistic depolymerization steps involved: milling at higher frequencies causes higher impact forces and thereby drives mechano-radicals further apart, making inter-radical reactions like instantaneous recombination and disproportionation reactions less likely,^39,40^ and promoting direct scission or radical transfer reactions which could alter the observed selectivity patterns. In addition, methane is likely produced *via* highly reactive methyl radicals (Scheme 1) whose formation is energetically challenging,^41^ especially compared to the more readily occurring β-scission leading to propene.^42^ Methane formation is therefore only observed at sufficiently high frequencies and impact forces which can activate mechano-radicals by increasing local energy densities enough to overcome significant energy barriers.

The different sensitivities of products with respect to the exponent in *f*_mill_ offer a path to obtain desired products more effectively. Especially propene yields can be boosted by increasing the milling frequency due to the large absolute amount and high exponent in *f*_mill_ (5.42). This is an interesting option for increasing the selectivity of an industrial process and changing the product distribution solely by changing the forces involved, for example by increasing the milling frequency or sphere size.

In the following, we apply the modified Zhurkov model to a variation of milling frequency and the effect we observed on small hydrocarbon productivity. Similarly as for the density series, using eqn (10) we use *σ* = *cf*_mill_^2/3^, with *c* being the proportionality constant. While the Zhurkov equation such as given in eqn (12) is valid for constant collision rates, it needs to be refined to reflect the increasing number of collisions with increasing milling frequency. More specifically, the number of chain cleavage events represented by 
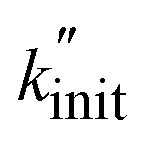
 is not only dependent on the inherent bond cleavage rate constant *k*, but also linearly scales with the number of relevant impacts, expressed *via* the collision frequency *ṅ*_coll_. As discussed before, we assume that *ṅ*_coll_ scales linearly with *f*_mill_, and therefore obtain eqn (16) with the definition in eqn (17).16
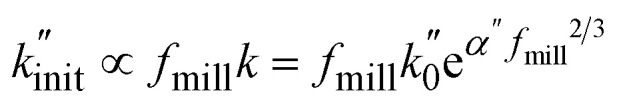
17
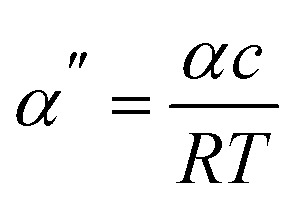


We fitted the 
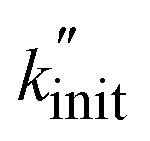
 values obtained from varying *f*_mill_ to eqn (16). The relationship qualitatively captures the data (Fig. 2E), showing that the Zhurkov model can effectively be applied to both the density as well as the frequency series. To check for consistency among the two fits, we calculated the activation volume *α* obtained by fitting the modified Zhurkov relationship to the density series with the one obtained from fitting it to the frequency series, assuming a temperature of 40 °C. The two values only differ by a factor of two, with *α* = 1.99 × 10^−3^ m^3^ mol^−1^ obtained from the density series and *α* = 1.00 × 10^−3^ m^3^ mol^−1^ for the frequency series. Due to the better quality of the data and the truly exponential relationship for the frequency series, we propose using the *α* value obtained from this series, *i.e.*, *α* = 1.00 × 10^−3^ m^3^ mol^−1^, until a more precise and unified value is available. We calculated the value obtained from Zhurkov^17^ to be *α* = 2.18 × 10^−4^ m^3^ mol^−1^, which is an order of magnitude lower than the value obtained by us (see Section S4† for details of the calculation).

### Role of sphere number and sphere size

We investigated the number of grinding spheres *n*_spheres_, using between 1 and 19 ZrO_2_ spheres with a diameter of 10 mm, shaking at 30 Hz. While milling with 1 sphere leads to virtually no propene generated, increasing propene formation rates are observed using more grinding spheres (see Fig. 2F and S4† for propene flows and Fig. S5† for cumulative yields, see Fig. S6† for different numbers of WC and Al_2_O_3_ spheres). Using more grinding spheres increases the number of collision events between spheres and plastic material resulting in more chain cleavage and ultimately small molecule formation. However, a limit is reached when filling the 25 ml container with *ca.* 9–11 spheres (Fig. 2F). While adding more grinding spheres increases the number of collisions, it restricts the free movement of other grinding spheres and the exchange of kinetic energy with the surrounding material. Therefore, adding more than the optimal amount of grinding spheres leads to a decline in sustained propene formation rates after 60 min due to the reduced void space in the jar.

We investigated the size of grinding spheres using one steel sphere with a diameter of 10 or 15 mm, shaking at 30 Hz. Milling with one 10 mm steel sphere produces almost no propene (Fig. S7†), like observed for ZrO_2_. Increasing the diameter to 15 mm increases small hydrocarbon yields significantly due to the higher impact forces of the larger and heavier grinding sphere and a resulting boost in chain cleavage activity.

### Role of plastic filling degree

**Fig. 3:**
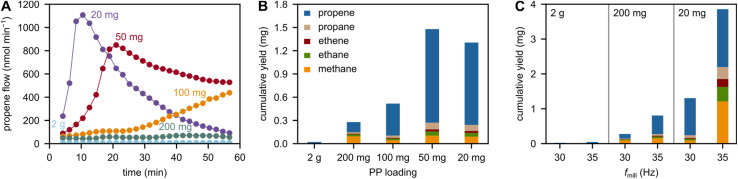
Variation of plastic filling degree. (A) Propene flow during milling of 20, 50, 100, 200, and 2000 mg model PP at 30 Hz with 5 ZrO_2_ grinding spheres (10 mm diameter). The declining propene flow after peak activity when milling with 20 mg PP is due to the low amount of polymer available for collisions at longer milling times. (B) Cumulative C_1–3_ hydrocarbon yields obtained after 1 h of milling 20, 50, 100, 200, and 2000 mg model PP at 30 Hz with 5 ZrO_2_ grinding spheres (10 mm diameter). (C) Cumulative C_1–3_ hydrocarbon yields obtained after 1 h of milling 20, 200, and 2000 mg model PP at 30 and 35 Hz with 5 ZrO_2_ grinding spheres (10 mm diameter).

We investigated the influence of the plastic filling degree by adjusting the mass of PP while keeping the number of grinding spheres and volume of container constant. Milling at lower plastic filling degrees has a large effect on the observed propene flows (Fig. 3A) and cumulative C_1–3_ hydrocarbon yields (Fig. 3B). At lower plastic filling degrees, more direct contact between the plastic and grinding spheres is possible. This leads to a mitigation of the cushioning effect, where plastic material decelerates the grinding spheres before compressing the plastic enough to induce chain cleavage. This cushioning ultimately leads to a loss of force transfer to the polymer chains. In its absence, however, chain cleavage is enhanced and higher rates of small hydrocarbon formation are observed.

The mitigation of the cushioning effect by low plastic filling degrees has been used to maximize monomer yields in the mechano-chemical recycling literature.^20,21^ High relative yields are often only accessible using very low plastic loadings and high shaking frequencies. This is also the case when milling PP (Fig. 3C). However, the suitability of such settings in a realistic recycling scenario is questionable. Ball milling is energy-intense, and when only small fractions of plastic are loaded into the milling container at a time, the absolute yield and conversion levels suffer significantly. In addition, such settings lower the selectivity to the desired monomer propene in the case of milling PP (see 20 mg at 35 Hz in Fig. 3C, see also Fig. 2B for 2 g). Moreover, we believe that there are boundaries to increasing yield by decreasing the plastic filling degree. Such boundaries can be caused by a decrease in collision efficiency at lower plastic filling degrees, such as observed for poly(α-methyl styrene).^20^ Furthermore, milling at such low loadings causes very high wear on the grinding materials (Fig. S8†). For this reason, the ball mill producer Retsch, for example, advises against filling less than a third of the container volume with plastic material, which would translate to grams of material rather than the milligrams commonly used. In our case, milling 20 mg model PP with ZrO_2_ spheres for 60 min at 35 Hz led to heavy abrasion of the milling tools, and prominent ZrO_2_ signals in the X-ray diffraction pattern of the residue (Fig. S9†).

While high-yield experiments showcase the potential of mechano-chemistry as a recycling strategy, milling at low plastic filling degrees is not sustainable from an energy and a material perspective. Thus, future work should focus on ways to increase hydrocarbon productivity at higher loadings to reach relevant absolute yields, for example by maximizing forces or using catalysts.

### Role of milling temperature

**Fig. 4:**
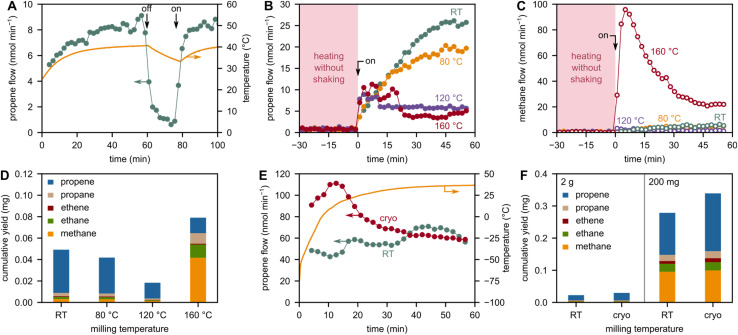
Effect of temperature on product formation during milling of PP. (A) Propene flow and external container temperature during milling of 2 g model PP at 30 Hz with 5 ZrO_2_ grinding spheres (10 mm diameter). The agitation was turned on and off at the indicated points in time. Propene (B) and methane (C) flows during milling of 2 g high molecular weight (HMW) PP at 30 Hz with 5 steel grinding spheres (10 mm diameter) at RT and 80, 120, and 160 °C. (D) Cumulative C_1–3_ hydrocarbon yields obtained after 1 h of milling 2 g HMW PP at 30 Hz with 5 steel spheres (10 mm diameter) at RT and 80, 120, and 160 °C. (E) Propene flow and external container temperature during milling of 200 mg model PP at 30 Hz with 5 ZrO_2_ grinding spheres (10 mm diameter) with and without pre-cooling in liquid nitrogen. (F) Cumulative C_1–3_ hydrocarbon yields obtained after 1 h of milling 200 mg and 2 g model PP at 30 Hz with 5 ZrO_2_ grinding spheres (10 mm diameter) with and without pre-cooling in liquid nitrogen.

During milling of 2 g model PP with 5 ZrO_2_ grinding spheres at 30 Hz, the bulk/container temperature rises to *ca.* 40 °C due to friction and dissipation of mechanical energy (Fig. 4A). When the milling is turned off, the small molecule flow stops immediately, while the temperature declines only slightly. We, therefore, believe that this bulk heating effect is not the cause for the formation of small hydrocarbons. On the other hand, the Zhurkov equation (eqn (1)) indicates a temperature dependency of chain cleavage rates. Furthermore, a change in temperature can alter the material properties of polymers significantly, and therefore also the milling efficiency and local stress concentrations in the material. To investigate the effect of milling temperature on the formation of small hydrocarbons, we milled PP at high (up to 160 °C) and low temperatures (starting at −196 °C).

We performed ball milling experiments at elevated temperatures by heating the container at 80, 120, and 160 °C for 1 h and then starting the shaking. Prior to milling, no C_1–3_ products are observed. Significant product evolution begins with the start of milling in all cases. Interestingly, increasing the milling temperature from room temperature (RT) to 80 to 120 °C decreases the yields of all products, such as propene (Fig. 4B). While the Zhurkov equation would suggest higher chain cleavage rates (and therefore also higher product formation) if the temperature is increased (at constant stress), we believe that this is counteracted by a less effective concentration of stress at single bonds. We believe that the temperature-induced softening of the plastic material leads to less effective milling in terms of force transferal,^43^ resulting in less chain cleavage and therefore a lower concentration of mechano-radical intermediates. Within the framework of our model, this can be rationalized by a decline in Young's modulus as the temperature increases,^44^ which leads to a lower stress and therefore less chain cleavage according to eqn (10) and (1). While higher temperatures cause, therefore, lower concentrations of mechano-radical intermediates, the reactivity of a single intermediate to form depolymerization products rises with temperature due to kinetic and thermodynamic reasons.^25^ To observe high yields of small hydrocarbons, both chain cleavage and depropagation steps have to be sufficiently fast and in balance with one another. However, it seems like increasing the temperature up to 120 °C does not accelerate depropagation rates enough to make up for the decrease in chain cleavage rates. Contrarily, increasing the temperature even further to 160 °C melts the PP but increases yields again, and the higher reactivity of radicals seems to make up for lower chain cleavage rates due to less effective force transferal. In addition, a combination of mechano- and thermo-chemical cracking seems plausible at these high temperatures. However, mostly over-cracking to methane is observed, rather than propene as the desired product of direct depolymerization (Fig. 4C and D). This shift in selectivity could be rooted in differences with respect to radical stability (primary *vs.* secondary *vs.* tertiary) at higher compared to lower temperatures.

To counteract the softening of PP at high temperatures, we performed experiments at low temperature by pre-cooling the container in liquid nitrogen (−196 °C) immediately before starting to shake. This cools PP below its glass transition temperature (−10 °C) and makes it glassy/brittle. Compared to milling at RT, we observed increased formation rates of propene and other products for an initial phase of milling which is likely caused by the increased chain cleavage rates at low temperatures (Fig. 4E and F). It has been shown that the glass transition temperatures in amorphous polymers are important descriptors for chain cleavage activity during ball milling,^22^ and the higher chain cleavage rates at cryogenic conditions seem to make up for lower depropagation rates in terms of product formation. We furthermore confirmed the preference for chain cleavage at cryogenic conditions compared to RT using electron spin resonance spectroscopy (Fig. S10†). After the initial cryogenic milling phase, however, the container temperature rises quickly above the *T*_g_ of PP due to frictional and ambient heating. Therefore, continuous cooling would be necessary to achieve a lasting effect. Although the industrial applicability of such a cryo-milling strategy is questionable, milling should at least be performed below the melting point of plastics (160 °C for PP). On the other hand, ideal operating temperatures are high enough to allow for the volatilization of desired products to remove them from the reaction system, which is important to drive depolymerization.

## Conclusions

Mechano-chemical degradation of polymers is a usually unwanted phenomenon in plastic production, but is currently investigated as a selective chemical recycling strategy. We showed the influence of milling parameters on the formation of small hydrocarbons from polypropylene. Generally, maximizing impact forces by using dense grinding spheres, high milling frequencies, and low plastic filling degrees increases hydrocarbon productivity significantly. We found that the role of grinding sphere density and frequency can be effectively captured by the polymer mechano-chemical Zhurkov equation which dictates an exponential effect of induced stress on backbone cleavage. Heavier spheres possess more kinetic energy which can be transferred to the plastic material. Increasing kinetic energy by faster milling is likewise effective, with total yields scaling with the milling frequency to the power of 5.51. High relative yields can be obtained by milling at low plastic filling degrees and high frequencies, causing more direct and hard contacts between grinding tools and plastic material. Especially the combination low plastic filling degrees and high frequencies, however, causes severe wear on the grinding materials. This mode of operation is not sustainable from an energy and material perspective, despite being often used as examples to prove the viability of mechano-chemical recycling strategies. Other ways of improving the yields *via*, *e.g.*, catalytic approaches have to be investigated, besides novel reactor geometries allowing for increased mechanical force transferal while minimizing wear on the grinding tools and maximizing material throughput. High temperatures during mechano-chemical recycling of polyolefins should be avoided as they lead to softening of plastic and less chain cleavage, as well as over-cracking to methane in more extreme cases. Cryogenic milling can increase yields by making plastic more brittle.

## Data availability

Data for this article, including gas chromatography, electron spin resonance, and X-ray diffraction data as well as analysis code are available *via* the Yoda repository at https://doi.org/10.24416/UU01-VCVD2Z.

## Conflicts of interest

There are no conflicts to declare.

## Supplementary Material

MR-002-D4MR00098F-s001
