# Supplementary material for: Influence of ball milling parameters on the mechano-chemical conversion of polyolefins
Source: RSC Mechanochem. 2024 Dec 18;2(2):263–72. doi: 10.1039/d4mr00098f (PMC11696860; doi:10.1039/d4mr00098f)
Supplement: MR-002-D4MR00098F-s001 [file MR-002-D4MR00098F-s001.pdf]

Supplementary Information (SI) for RSC Mechanochemistry.
This journal is © The Royal Society of Chemistry 2025

Supporting Information for

Influence of ball milling parameters on the mechano-chemical conversion of polyolefins

Adrian H. Hergesell, Claire L. Seitzinger, Justin Burg, Renate J. Baarslag, Ina Vollmer*

Inorganic Chemistry and Catalysis, Institute for Sustainable and Circular Chemistry, Utrecht University, Universiteitsweg 99, 3584 CG, Utrecht, The Netherlands. E-mail: i.vollmer@uu.nl

Supporting Information

S1 – Experimental details

We investigated the mechano-chemical conversion of three plastic materials into small hydrocarbons. Polypropylene (PP) and polyethylene (PE) were commercially available polymer products (**Table S1**).

In a typical experiment, model PP or ultra high molecular weight (UHMW) PE and potential additives were loaded into a modified tungsten carbide ball milling container (25 ml, Retsch) and grinding spheres of 10 mm diameter were added (**Table S4**). A Teflon seal was used and the container was closed with a wrench and shaken for a specific time at a specific frequency. A Retsch MM400 mixer mill was used for experiments in which the number of ZrO_2 grinding spheres was varied. All other experiments were performed on an equivalent Retsch MM500 vario mixer mill. After collecting the milled material for potential further analyses, the container was cleaned with water and acetone, and dried at 110 °C for > 2 h.

For ball milling experiments at 80, 120, and 160 °C, the container was loaded with 2 g high molecular weight (HMW) PP and wrapped in a flexible glass yarn–insulated heating cable (Horst, 1.0 m, 100 W). A thermocouple was attached directly to the container, and both the heating wire and the thermocouple were wrapped with woven fiberglass insulation tape. All materials were secured with heat-resistant adhesive tape. Prior to milling, the desired temperature was set and the formation of volatile hydrocarbons was monitored during a period of ca. 60 min.

For ball milling experiments starting at cryogenic temperatures, the container was loaded with model PP, flushed with 12.5 ml min^{-1} N_2 and pre-cooled in a liquid nitrogen bath for 10 min. Milling was started directly after removing the container from the bath and attaching it to the ball mill.

For the continuous analysis of gaseous hydrocarbon products, the container was equipped with 1/8″ Swagelok connections, after drilling holes via electrical discharge machining. A continuous flow of 12.5 ml min^{-1} N_2 was used to elute hydrocarbons and functioned as an internal standard. Products were analyzed on an online Global Analyser Solutions gas chromatograph (GC). A thermal conductivity detector (TCD) coupled to a 2 m × 0.32 mm Rtx-1, 3.0u and a 3 m × 0.32 mm Carboxen1010 column was used for the detection of N_2 and H_2. For the detection of hydrocarbons, a flame ionization detector (FID) was utilized (C_{1-3}: 3 m × 0.32 mm Rtx-1, 3u column and 15 m × 0.32 mm Al_2O_3/Na_2SO_4 column; C_{4-7}: 2 m × 0.28 mm MXT-1, 1u column and 14 m × 0.28 mm MXT-1, 1u column; C_{5-10}: 2 m × 0.28 mm MXT-1, 0.5u column and 15 m × 0.28 mm MXT-1, 0.5u column). To counter any potential changes in total volumetric flow ($F_{\mathrm{total},i} = \frac{F_{\mathrm{N_2}}}{y_{\mathrm{N_2},i}}$) caused by the formation of products, the constant flow of N_2 ($F_{\mathrm{N_2}}$ = 12.5 ml min^{-1} = const.) was used as an internal standard. We used **Eq. 1** to determine the molar concentration of N_2 at each injection during the run ($y_{\mathrm{N_2},i}$). This equation uses the average of the peak areas ($A_{\mathrm{N_2},i}$) of the first three injections prior to start of the reaction after the N_2 flow had stabilized.

$$y_{\mathrm{N_2},i} = \frac{A_{\mathrm{N_2},i}}{\frac{\sum_{i=-2}^{i=0} A_{\mathrm{N_2},i}}{3}} \cdot \ y_{N_{2,0}} \qquad (1)$$

We used **Eq. 2** to calculate the molar carbon flow of each hydrocarbon (C_xH_y) by using its carbon number x.

$$F_{\mathrm{C}_x\mathrm{H}_y,i} = y_{\mathrm{C}_x\mathrm{H}_y,i} \cdot F_{\mathrm{total}} \cdot x \tag{2}$$

Each hydrocarbon's concentration ($y_{\mathrm{C}_x\mathrm{H}_y,i} = \frac{A_{\mathrm{C}_x\mathrm{H}_y,i}}{CF_{\mathrm{C}_x\mathrm{H}_y,i}}$) was calculated by the ratio of peak area of this hydrocarbon ($A_{\mathrm{C}_x\mathrm{H}_y,i}$) and a calibration factor ($CF_{\mathrm{C}_x\mathrm{H}_y,i}$). The calibration factor was determined by injecting a gas mixture with known content. $CF_{\mathrm{C}_x\mathrm{H}_y,i} = CF_{\mathrm{C}} \cdot x$, where CF_{C} is the calibration factor normalized by carbon number, which was determined by calibration with a mixture of methane, ethane, propane, butane, heptane and hexane. The signal response of an FID is approximately proportional to the carbon number of the hydrocarbon.

We used **Eq. 3** to determine the cumulative yield of a certain hydrocarbon. This equation integrates its molar flow over time (with $M_{\mathrm{C}_x\mathrm{H}_y}$ as the hydrocarbon's molecular weight).

$$Y_{\mathrm{C}_x\mathrm{H}_y}\,[\mathrm{g}] = \frac{M_{\mathrm{C}_x\mathrm{H}_y}}{x} \cdot \int_0^{t_{\mathrm{final}}} F_{\mathrm{C}_x\mathrm{H}_y,i}\,\mathrm{d}t \tag{3}$$

Electron spin resonance (ESR) measurements were performed in continuous wave mode on an X-band Bruker EMXplus instrument. For spin trapping experiments on 5 g model PP, 100 mg bis(2,2,6,6-tetramethyl-4-piperidyl) sebacate (Sigma-Aldrich) was added to an un-modified milling container under air together with 5 WC grinding spheres of 10 mm diameter. The container was closed, and pre-cooled directly before milling by submerging it in liquid nitrogen for 10 min and milled for 5 min at 30 Hz. After milling, ca. 30 mg of milled material were filled into a quartz tube and measured at room temperature at a microwave frequency of ca. 9.4 GHz and a modulation frequency of 100 kHz.

X-ray diffraction (XRD) was performed on a Bruker 2D PHASER instrument, using Cu K_α radiation (λ = 1.54056 Å) and a LYNXEYE-2 detector. Samples were analyzed between 10 and 70° 2θ with a step size of 0.1°, a step time of 1 s and a sample rotation of 15 rpm.

Table S1. Used plastic materials and sources, shapes, number average molar mass (M_n) and weight average molar mass (M_w) given by suppliers.

Denoted as	Source	Product	Shape	M_n (g mol^{-1})	M_w (g mol^{-1})
model PP	Sigma-Aldrich	428116	pellets	5,000	12,000
HMW PP	Ducor	DuPure G72TF	powder		
UHMW PE	Sigma-Aldrich	429015	powder		3,000,000–6,000,000

Table S2. Mechanical properties of PP, ZrO_2, Al_2O_3, Fe, and WC: Density ρ, Young's modulus E, Poisson's ratio μ.

	ρ (g cm^{-3})	E (GPa)	μ (–)
PP	0.9[a]	1.847[1]	0.421[1]
ZrO_2	6.05[b]	209[2]	0.322[2]
Al_2O_3	3.8[b]	390[2]	0.242[2]
WC	14.8[b]	708[3]	0.2343[3]
Fe	7.835[b]	194–206[4]	0.294–0.301[5]

[a]Supplier value (Sigma-Aldrich). [b]Supplier value (Retsch).

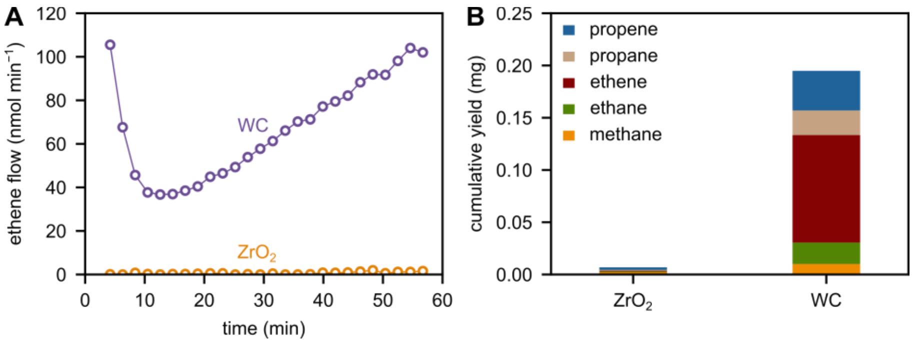

Fig. S1. (A) Ethene flow during milling of 2 g UHMW PE at 30 Hz with 5 ZrO_2 and WC grinding spheres (10 mm diameter). **(B)** Cumulative C_{1-3} hydrocarbon yields obtained after 1 h of milling 2 g UHMW PE at 30 Hz with 5 ZrO_2 and WC grinding spheres (10 mm diameter).

Table S3. Sphere material, sphere radius r, mass of an individual sphere m, number of spheres $n_{spheres}$, milling frequency f_{mill} and resulting total energy E_{total}, as calculated according to Jafter *et al.*[6] We used a jar diameter of 27 mm, a jar length of 53 mm, an oscillation amplitude of 15 mm, a ball diameter of 10 mm and a milling time of 60 min.

Material	r (mm)	m (g)	$n_{spheres}$	f_{mill} (Hz)	E_{total} (kJ)
Al_2O_3	5	1.99	5	30	42.5
ZrO_2	5	3.17	5	30	67.8
Fe	5	4.10	5	30	87.6
WC	5	7.75	5	30	165.6
ZrO_2	5	3.17	5	25	39.2
ZrO_2	5	3.17	5	26	44.1
ZrO_2	5	3.17	5	27	49.4
ZrO_2	5	3.17	5	28	55.1
ZrO_2	5	3.17	5	29	61.2
ZrO_2	5	3.17	5	30	67.8
ZrO_2	5	3.17	5	31	74.8
ZrO_2	5	3.17	5	32	82.2
ZrO_2	5	3.17	5	33	90.2
ZrO_2	5	3.17	5	34	98.6
ZrO_2	5	3.17	5	35	107.6
WC	5	7.75	5	20	49.1
WC	5	7.75	5	22	65.3
WC	5	7.75	5	24	84.8
WC	5	7.75	5	26	107.8
WC	5	7.75	5	28	134.7
WC	5	7.75	5	30	165.6
ZrO_2	5	3.17	1	30	13.7
ZrO_2	5	3.17	3	30	40.9
ZrO_2	5	3.17	5	30	67.8
ZrO_2	5	3.17	7	30	93.6
ZrO_2	5	3.17	9	30	117.7
ZrO_2	5	3.17	11	30	139.4
ZrO_2	5	3.17	13	30	157.7
ZrO_2	5	3.17	15	30	171.6
ZrO_2	5	3.17	17	30	180.0
ZrO_2	5	3.17	19	30	181.8

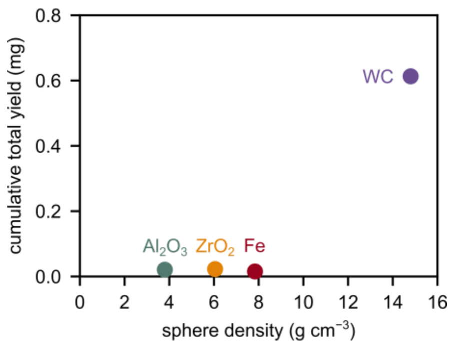

Fig. S2. Cumulative total C_{1-3} hydrocarbon yields obtained after 1 h of milling 2 g model PP at 30 Hz with 5 Al_2O_3, ZrO_2, Fe, and WC grinding spheres (10 mm diameter) vs. sphere density.

S2 – Calculation of container velocity

We assume a sinusoidal movement equation of the container where f_{mill} is the milling frequency and x_{max} the shaking amplitude.

$$x(t) = x_{\mathrm{max}} \cdot \sin(2\pi f_{\mathrm{mill}} t) \tag{4}$$

The container velocity $\dot{x}(t)$ is obtained by derivatization of the movement equation.

$$\dot{x}(t) = x_{\mathrm{max}} \cdot 2\pi f_{\mathrm{mill}} \cdot \cos(2\pi f_{\mathrm{mill}} t) \tag{5}$$

The velocity equation can be rewritten as a function of $\dot{x}_{\mathrm{max}}$, which is the amplitude of $\dot{x}(t)$ and the maximum velocity of the container. $\dot{x}_{\mathrm{max}}$ is linearly dependent on f_{mill}.

$$\dot{x}(t) = \dot{x}_{\mathrm{max}} \cdot \cos(2\pi f_{\mathrm{mill}} t) \tag{6}$$

$$\dot{x}_{\mathrm{max}} = x_{\mathrm{max}} \cdot 2\pi f_{\mathrm{mill}} \tag{7}$$

We determined x_{max} to be ca. 1.15 cm. Therefore, at 30 Hz, $\dot{x}_{\mathrm{max}}$ is 2.17 m/s. At 27.5 Hz, $\dot{x}_{\mathrm{max}}$ would be 1.99 m/s, which is close to results obtained with high-speed video analysis.[7]

The acceleration equation $\ddot{x}(t)$ can be obtained by derivatization of the velocity equation.

$$\ddot{x}(t) = -x_{\mathrm{max}} \cdot (2\pi f_{\mathrm{mill}})^2 \cdot \sin(2\pi f_{\mathrm{mill}} t) \tag{8}$$

The acceleration equation can be rewritten as a function of $\ddot{x}_{\mathrm{max}}$, which is the amplitude of $\ddot{x}(t)$ and the maximum acceleration of the container. $\ddot{x}_{\mathrm{max}}$ is quadratically dependent on f_{mill}. We define a_{max} as the absolute of $\ddot{x}_{\mathrm{max}}$.

$$\ddot{x}(t) = \ddot{x}_{\mathrm{max}} \cdot \sin(2\pi f_{\mathrm{mill}} t) \tag{9}$$

$$\ddot{x}_{\mathrm{max}} = -x_{\mathrm{max}} \cdot (2\pi f_{\mathrm{mill}})^2 \tag{10}$$

$$a_{\mathrm{max}} = |\ddot{x}_{\mathrm{max}}| \tag{11}$$

S3 – Steady state approximation

As only the gas phase product formation can be monitored, to calculate the chain cleavage rate, we use a simple mechanistic model consisting of initiation, termination, and depolymerization, and the steady state approximation. P_m denotes polymer material, $\mathrm{P}_n^{\cdot}$ denotes polymer material with a radical functionality, and M denotes monomer molecules, namely propene, formed due to depolymerization. k_{init} is the rate of heterolytic backbone cleavage leading to two $\mathrm{P}_n^{\cdot}$. k_{term} is the termination rate when two $\mathrm{P}_n^{\cdot}$ react to form polymer material without radical functionality, for example via combination or disproportionation. k_{depol} is the depolymerization rate and describes processes that generate small hydrocarbons from $\mathrm{P}_n^{\cdot}$, without the latter losing its radical functionality.

$$\mathrm{P}_m \xrightarrow{k_{\mathrm{init}}} 2\ \mathrm{P}_n^{\cdot} \tag{12}$$

$$2\ \mathrm{P}_n^{\cdot} \xrightarrow{k_{\mathrm{term}}} \mathrm{P}_m \tag{13}$$

$$\mathrm{P}_n^{\cdot} \xrightarrow{k_{\mathrm{depol}}} \mathrm{P}_n^{\cdot} + \mathrm{M} \tag{14}$$

Expressions for the rates of these reactions are:

$$r_{\mathrm{init}} = k_{\mathrm{init}} \cdot [\mathrm{P}_m] \tag{15}$$

$$r_{\mathrm{term}} = 2 \cdot k_{\mathrm{term}} \cdot [\mathrm{P}_n^{\cdot}]^2 \tag{16}$$

$$r_{\mathrm{depol}} = k_{\mathrm{depol}} \cdot [\mathrm{P}_n^{\cdot}] = \frac{\mathrm{d[M]}}{\mathrm{d}t} \tag{17}$$

As an approximation of steady state, we assume $r_{\mathrm{init}} = r_{\mathrm{term}}$, and derive the following expression:

$$k_{\mathrm{init}} \cdot [\mathrm{P}_m] = 2 \cdot k_{\mathrm{term}} \cdot [\mathrm{P}_n^{\cdot}]^2 \tag{18}$$

$$\Leftrightarrow k_{\mathrm{init}} = \frac{2 \cdot k_{\mathrm{term}} \cdot [\mathrm{P}_n^{\cdot}]^2}{[\mathrm{P}_m]} \tag{19}$$

Using the definition of r_{depol} with $[\mathrm{P}_n^{\cdot}] = \frac{\frac{\mathrm{d[M]}}{\mathrm{d}t}}{k_{\mathrm{depol}}}$ we obtain:

$$k_{\mathrm{init}} = \frac{2 \cdot k_{\mathrm{term}} \cdot \left(\frac{\mathrm{d[M]}}{\mathrm{d}t}\right)^2}{k_{\mathrm{depol}}^2 \cdot [\mathrm{P}_m]} \tag{20}$$

Since P_m is present in large excess, $[\mathrm{P}_m]$ can be assumed to be constant. We define a combined rate constant $\kappa = \frac{2 \cdot k_{\mathrm{term}}}{k_{\mathrm{depol}}^2 \cdot [\mathrm{P}_m]}$, and obtain:

$$k_{\mathrm{init}} = \kappa \cdot \left(\frac{\mathrm{d[M]}}{\mathrm{d}t}\right)^2 \tag{21}$$

We obtain k'_{init} as a modified version of k_{init}.

$$k'_{\mathrm{init}} = \frac{k_{\mathrm{init}}}{\kappa} = \left(\frac{\mathrm{d[M]}}{\mathrm{d}t}\right)^2 \tag{22}$$

Due to the nature of our reactor design, in which small molecules are continuously removed from the reaction environment, we do not use $\frac{\mathrm{d}[\mathrm{M}]}{\mathrm{d}t}$ to describe the kinetics. Instead, we use the stoichiometric amount of monomers $n(\mathrm{M})$ generated during the time t, and obtain k''_{init} as a surrogate for k'_{init} as the chain cleavage rate.

$$k''_{\mathrm{init}} = \left(\frac{n(\mathrm{M})}{t}\right)^2 \tag{23}$$

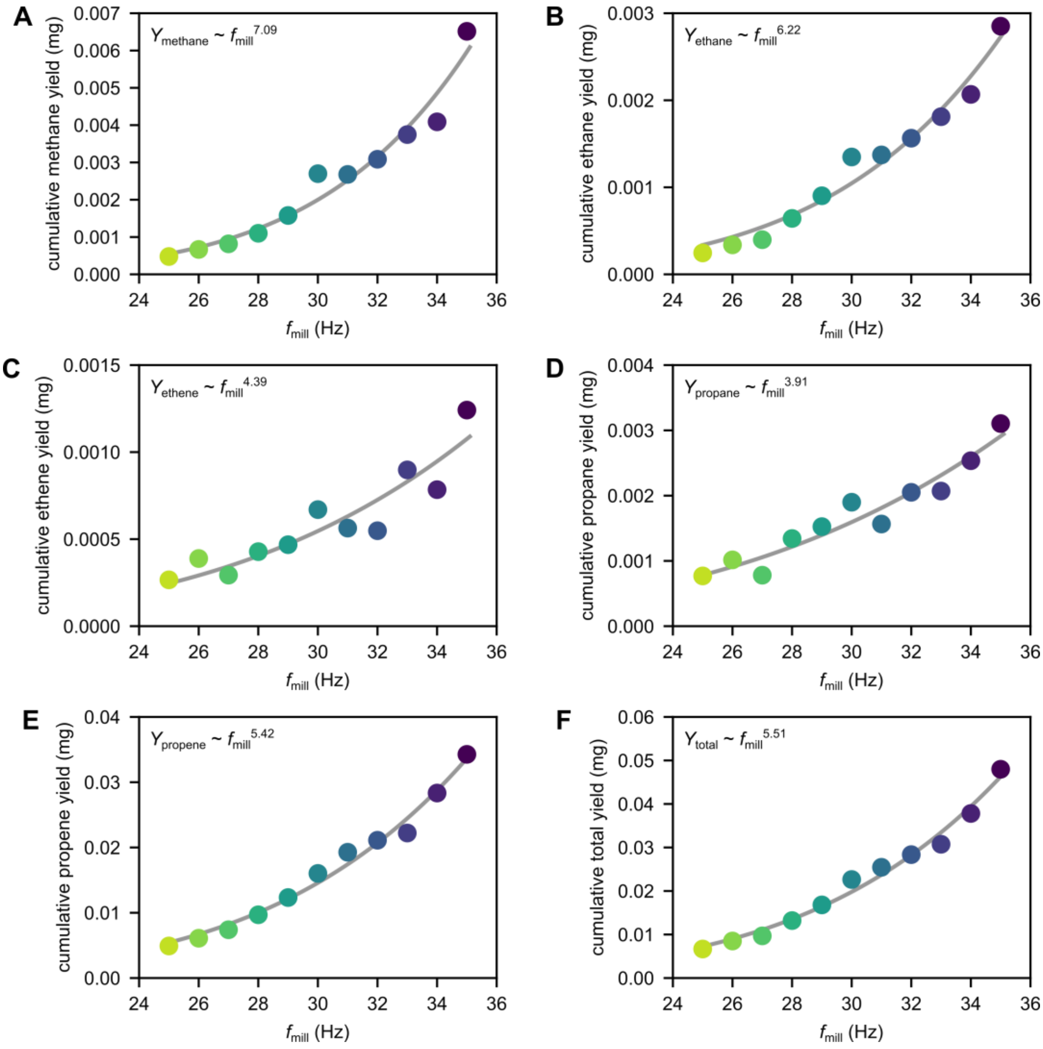

Fig. S3. Cumulative hydrocarbon yields of **(A)** methane, **(B)** ethane, **(C)** ethene, **(D)** propane, and **(E)** propene and **(F)** total cumulative yield obtained after 1 h of milling 2 g model PP at 25–35 Hz with 5 ZrO_2 grinding spheres (10 mm diameter), and fit of $\frac{Y_i}{\mathrm{mg}} = a_i \cdot \left(\frac{f_{\mathrm{mill}}}{\mathrm{Hz}}\right)^{x_i}$ for each product i. Y_i denotes the cumulative yield of i after milling.

S4 – Calculation of α values

We fitted the data for the variation of density in **Fig. 1C** according to **Eq. 14** (main text):

$$k''_{\text{init}} = 3.74 \cdot 10^{-16} \cdot e^{1.54 \cdot \rho^{\frac{1}{3}}} \left[\frac{\text{nmol}^2}{\text{s}^2}\right] \tag{24}$$

We therefore obtained:

$$\alpha' = \frac{\alpha b}{RT} = 1.54 \text{ m kg}^{-\frac{1}{3}} \tag{25}$$

We use $T = 273.14 + 40 \text{ K}$, and

$$b = 2^{\frac{5}{3}} (E^*)^{\frac{2}{3}} (r x_{\text{max}})^{\frac{1}{3}} f_{\text{mill}}^{\frac{2}{3}} \tag{26}$$

where E^* is calculated with the properties of PP and WC (**Table S2**) to obtain from the density series:

$$\alpha = 1.99 \cdot 10^{-3} \frac{\text{m}^3}{\text{mol}} \tag{27}$$

We fitted the data for the variation of frequency in **Fig. 2E** according to **Eq. 16** (main text):

$$k''_{\text{init}} = 2.42 \cdot 10^{-10} \cdot f_{\text{mill}} \cdot e^{1.46 \cdot f_{\text{mill}}^{\frac{2}{3}}} \left[\frac{\text{nmol}^2}{\text{s}^2}\right] \tag{28}$$

We, therefore, obtained:

$$\alpha'' = \frac{\alpha c}{RT} = 1.46 \text{ s}^{\frac{2}{3}} \tag{29}$$

We use $T = 273.14 + 40 \text{ K}$, and

$$c = 2^{\frac{5}{3}} (E^*)^{\frac{2}{3}} (r x_{\text{max}})^{\frac{1}{3}} \rho^{\frac{1}{3}} \tag{30}$$

where E^* is calculated with the properties of PP and ZrO_2 (**Table S2**) to obtain from the frequency series:

$$\alpha = 1.00 \cdot 10^{-3} \frac{\text{m}^3}{\text{mol}} \tag{31}$$

For PP, Zhurkov[8] reports $0.51 \frac{\text{kcal} \cdot \text{mm}^2}{\text{mol} \cdot \text{kg}}$ using the stress in $\frac{\text{kg}}{\text{mm}^2}$. We assume that the stress can be converted to Pa by multiplying with the earth gravitational acceleration of $g = 9.8 \frac{\text{m}}{\text{s}^2}$. We thus obtain the following for α according to Zhurkov:

$$\alpha = \frac{0.51 \frac{\text{kcal} \cdot \text{mm}^2}{\text{mol} \cdot \text{kg}} \cdot 4184 \frac{\text{J}}{\text{kcal}} 10^{-6} \frac{\text{m}^2}{\text{mm}^2}}{9.8 \frac{\text{m}}{\text{s}^2}} = 2.18 \cdot 10^{-4} \frac{\text{m}^3}{\text{mol}}$$

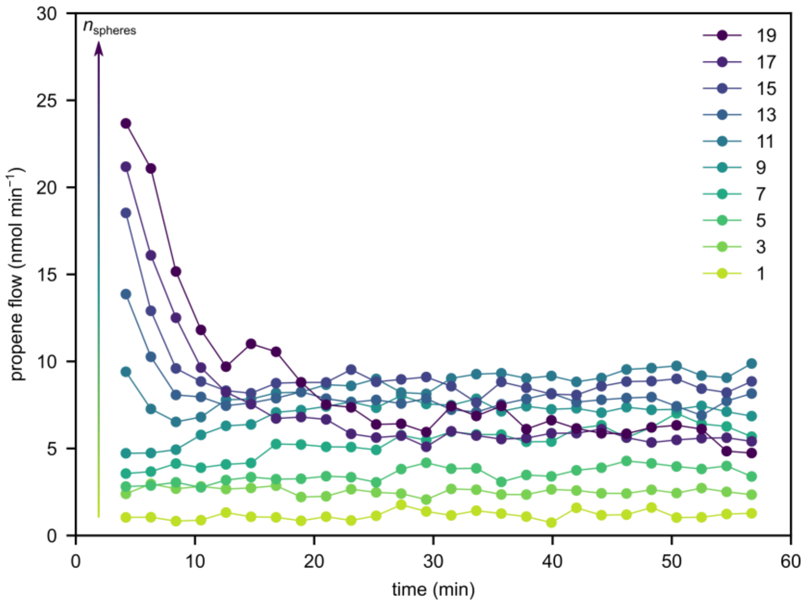

Fig. S4. Propene flow during milling of 2 g model PP at 30 Hz with 1–19 ZrO_2 grinding spheres (10 mm diameter).

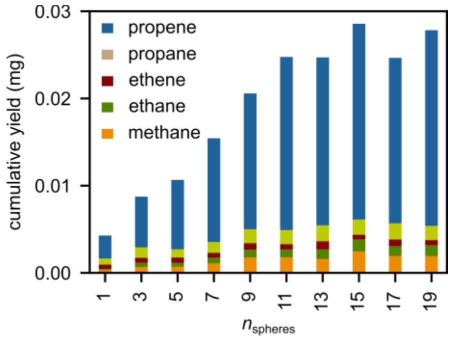

Fig. S5. Cumulative C_{1-3} hydrocarbon yields obtained after 1 h of milling 2 g model PP at 30 Hz with 1–19 ZrO_2 grinding spheres (10 mm diameter).

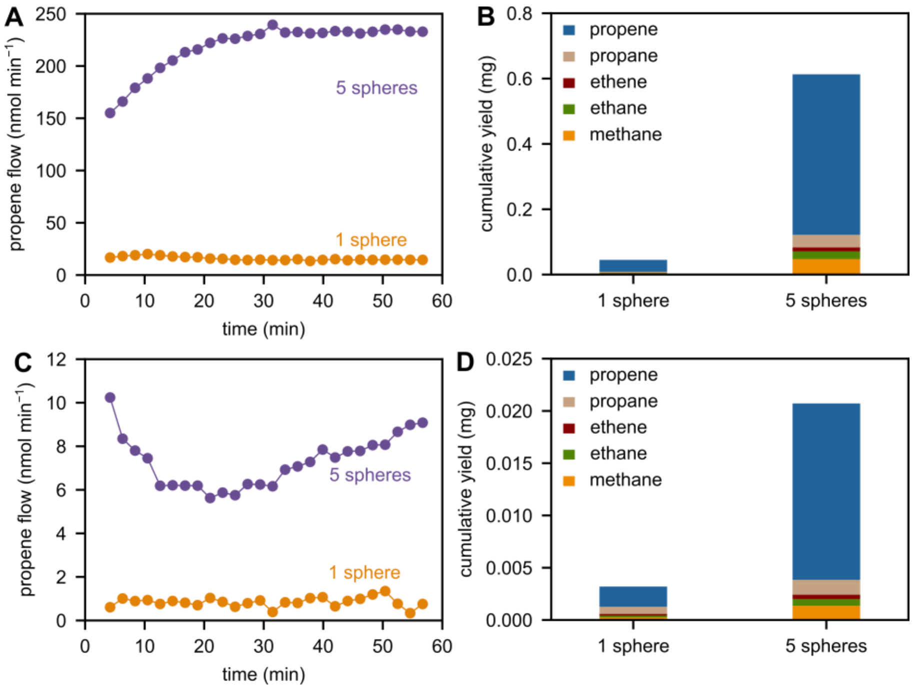

Fig. S6. (A) Propene flow during milling of 2 g model PP at 30 Hz with 1 and 5 WC grinding spheres (10 mm diameter). **(B)** Cumulative C_{1-3} hydrocarbon yields obtained after 1 h of milling 2 g model PP at 30 Hz with 1 and 5 WC grinding spheres (10 mm diameter). **(C)** Propene flow during milling of 2 g model PP at 30 Hz with 1 and 5 Al_2O_3 grinding spheres (10 mm diameter). **(D)** Cumulative C_{1-3} hydrocarbon yields obtained after 1 h of milling 2 g model PP at 30 Hz with 1 and 5 Al_2O_3 grinding spheres (10 mm diameter).

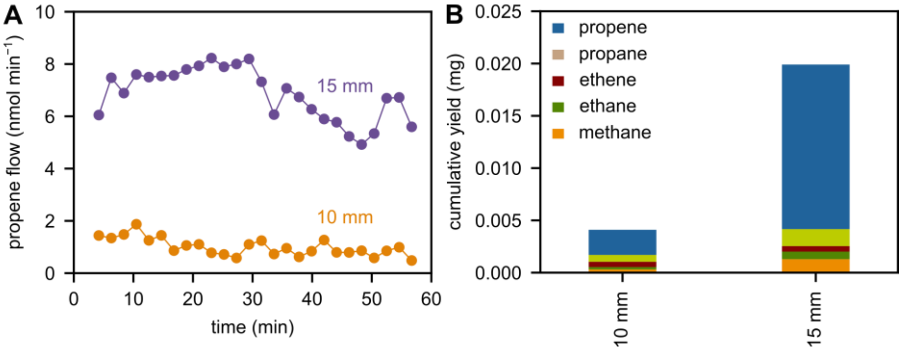

Fig. S7. (A) Propene flow during milling of 2 g model PP at 30 Hz with 1 steel grinding sphere (10 and 15 mm diameter). **(B)** Cumulative C_{1-3} hydrocarbon yields obtained after 1 h of milling 2 g model PP at 30 Hz with 1 steel grinding sphere (10 and 15 mm diameter).

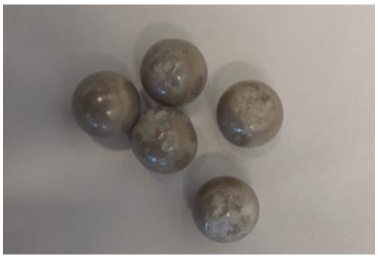

Fig. S8. Photograph of 5 damaged ZrO_2 grinding spheres (10 mm diameter) after repeated milling at 35 Hz with low plastic filling degrees of model PP.

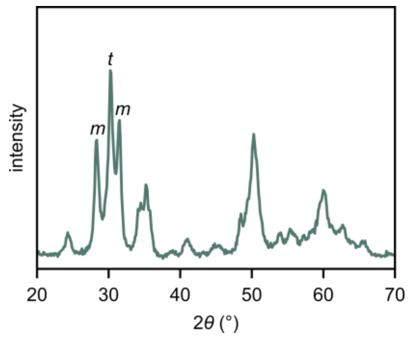

Fig. S9. X-ray diffractogram of milled residue after milling 20 mg model PP for 1 h at 35 Hz with 5 ZrO_2 grinding spheres (10 mm diameter). Some signals related to monoclinic (*m*) and tetragonal (*t*) ZrO_2 are indicated.

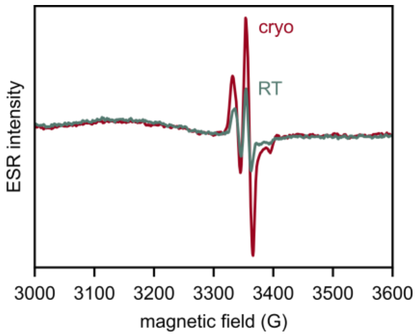

Fig. S10. Mass-normalized ESR spectra of a sample taken after milling 5 g model PP and 100 mg bis(2,2,6,6-tetramethyl-4-piperidyl) sebacate with 5 WC grinding spheres (10 mm diameter) for 5 min in air at 30 Hz, at RT and after cooling in liquid nitrogen. Radical species were stabilized by reacting with dioxygen and bis(2,2,6,6-tetramethyl-4-piperidyl) sebacate, ultimately leading to nitroxide radicals which are detected by ESR. The higher signal intensity and double integral after milling at cryogenic conditions vs. RT indicates more trapped radicals and therefore more chain cleavage due to the more brittle nature of PP below its T_g.

Table S4. Used griding spheres and their source.

Sphere material	Source	Used for
Fe	Retsch	all experiments
WC	Retsch	all experiments
Al_2O_3	InSolido Technologies	all experiments
ZrO_2	Zhonglong Materials	number variation
	Laarmann	all other experiments

List of symbols

A_{impact}	Impact radius
$a_{\max}$	Acceleration amplitude of ball mill container, $a_{\max} = \lvert\ddot{x}_{\max}\rvert$
b	Proportionality constant to connect σ and ρ via $\sigma = b\rho^{\frac{1}{3}}$
c	Proportionality constant to connect σ and f_{mill} via $\sigma = cf_{\mathrm{mill}}^{2/3}$
DKE	Dose of kinetic energy
E	Young's modulus
E^*	Reduced Young's modulus
E_{A}	Activation energy
E_{kin}	Impact kinetic energy of a grinding sphere
F_{impact}	Impact force
f_{mill}	Milling frequency
k	Rate constant for chain cleavage
k_0	Pre-exponential factor
k_0'	Modified pre-exponential factor
k_0''	Modified pre-exponential factor
k_{depol}	Depolymerization rate constant
k_{init}	Initiation rate constant
k_{init}''	Modified initiation rate constant
k_{term}	Termination rate constant
M	Monomer
m	Mass of a grinding sphere
n	Stoichiometric amount
$\dot{n}_{\mathrm{coll}}$	Collision frequency
n_{spheres}	Number of grinding spheres
P_m	Polymer material
$\mathrm{P}_n^{\cdot}$	Polymer material with a radical functionality,
R	Gas constant
RT	Room temperature
r	Sphere radius
r_{depol}	Depolymerization rate

r_{init}	Initiation rate
r_{term}	Termination rate
T	Temperature
t	Time
T_{c}	Ceiling temperature
T_{g}	Glass transition temperature
v	Impact velocity of a grinding sphere
x	Position of ball mill container
$\dot{x}$	Velocity of ball mill container
$\ddot{x}$	Acceleration of ball mill container
x_{max}	Shaking amplitude of ball mill container
$\dot{x}_{\mathrm{max}}$	Velocity amplitude of ball mill container
$\ddot{x}_{\mathrm{max}}$	Acceleration amplitude of ball mill container, $\lvert\ddot{x}_{\mathrm{max}}\rvert = a_{\mathrm{max}}$
Y	Yield
α	Activation volume
α'	Modified activation volume, $\alpha' = \frac{\alpha b}{RT}$
α''	Modified activation volume, $\alpha'' = \frac{\alpha c}{RT}$
κ	Combined rate constant
μ	Poisson’s ratio
ξ	Depth of indentation
ρ	Density of a grinding sphere
σ	Stress

References

1 M. Kumar, K. K. Gaur and C. Shakher, Measurement of Material Constants (Young's Modulus and Poisson's Ratio) of Polypropylene Using Digital Speckle Pattern Interferometry (DSPI), *J. Japanese Soc. Exp. Mech.*, 2015, **15**, s87–s91.

2 M. Borba, M. D. de Araújo, E. de Lima, H. N. Yoshimura, P. F. Cesar, J. A. Griggs and Á. Della Bona, Flexural strength and failure modes of layered ceramic structures, *Dent. Mater.*, 2011, **27**, 1259–1266.

3 R. R. Reeber and K. Wang, Thermophysical Properties of α-Tungsten Carbide, *J. Am. Ceram. Soc.*, 1999, **82**, 129–135.

4 I. Arrayago, K. J. R. Rasmussen and E. Real, Statistical analysis of the material, geometrical and imperfection characteristics of structural stainless steels and members, *J. Constr. Steel Res.*, 2020, **175**, 106378.

5 M. Spittel and T. Spittel, in *Metal Forming Data of Ferrous Alloys - deformation behaviour*, 2009, pp. 85–88.

6 O. F. Jafter, S. Lee, J. Park, C. Cabanetos and D. Lungerich, Navigating Ball Mill Specifications for Theory-to-Practice Reproducibility in Mechanochemistry, *Angew. Chem., Int. Ed.*, , DOI:10.1002/anie.202409731.

7 A. W. Tricker, G. Samaras, K. L. Hebisch, M. J. Realff and C. Sievers, Hot spot generation, reactivity, and decay in mechanochemical reactors, *Chem. Eng. J.*, 2020, **382**, 122954.

8 S. N. Zhurkov and V. E. Korsukov, Atomic mechanism of fracture of solid polymers, *J. Polym. Sci., Polym. Phys. Ed.*, 1974, **12**, 385–398.
